# Editorial: Tenascins – Key Players in Tissue Homeostasis and Defense

**DOI:** 10.3389/fimmu.2021.834353

**Published:** 2022-01-12

**Authors:** Kyoko Imanaka Yoshida, Kim S. Midwood, Gertraud Orend

**Affiliations:** ^1^Department of Pathology and Matrix Biology, Mie University Graduate School of Medicine, Tsu, Japan; ^2^Kennedy Institute of Rheumatology, University of Oxford, Oxford, United Kingdom; ^3^University Strasbourg, INSERM U1109, The Tumor Microenvironment Laboratory, Hôpital Civil, Institut d’Hématologie et d’Immunologie, Fédération de Médecine Translationnelle de Strasbourg (FMTS), 1 Place de l’Hopital, Strasbourg, France

**Keywords:** tenascins, extracellular matrix, inflammation, infection, cancer, fibrosis, tissue homeostasis, defense

Tenascin-C, -R, -X and -W are the four members of a family of large, multimodular, extracellular matrix molecules. By virtue of a large repertoire of binding partners, including other matrix molecules, soluble factors, and cell surface receptors, tenascins are key regulators of both tissue architecture and cell phenotype. This Research Topic looks back over the discovery of these matrix molecules over 40 years ago and highlight how our understanding of tenascin-related biology, and pathology, has exponentially progressed over this time. This issue also addressed how these molecules are being exploited for use in the diagnosis and treatment of inflammatory and fibrotic diseases, and cancer, in the context of immune checkpoint therapy and beyond.

One may ask for the justification of a Research Topic on *“Tenascins: Key Players in Tissue Homeostasis and Defense” in “Frontiers in Immunology”*. It has been some time now since the extracellular matrix has been considered merely an inert static mass of molecules that exists only to provide cells with support and stability. More recently has come the understanding of the key role that the matrix plays in general in directly controlling immune responses. In particular, the role of the tenascin family in actively communicating with cells to maintain tissue homeostasis, to signal perturbations in homeostasis and to orchestrate inflammatory and repair programmes to eliminate threat and restore homeostasis, is becoming ever more evident. Each tenascin family member exhibits a specifically restricted, largely non-overlapping, pattern of expression that is tightly controlled in healthy tissues, helping to create distinct microenvironmental niches that program localized cell behavior, for example in stem cell niches, in areas of high mechanical load, in connective tissue and in the perineural nets. The expression of these molecules is transiently upregulated in response to cellular stress and tissue injury, where they each play diverse roles in activating immune responses designed to restore homeostasis. However, misregulated expression of these molecules is associated with aberrant inflammation in inflammatory, fibrotic, and metabolic diseases, and cancer.

This Research Topic of 22 papers comprises 13 review articles and 9 original articles, co-authored by over 100 researchers in the field, that elaborate on both the history and the current state of the art in the fascinating story of the tenascins in the immune context. Indeed, the story actually starts a long way back in time. In this issue, Orend and Tucker showed that the first family member linked to a role in inflammation, tenascin-C (TNC) coevolved with adaptive immunity, and identify a “tool kit” used in cells regulating immunity and interacting with TNC. This raises the question whether the presence and conservation of TNC amongst vertebrates may be explained by its important role in immunity. There is an ever expanding literature on the mechanisms underpinning TNC-mediated inflammation following its identification as a damage associated molecular pattern (DAMP) that drives toll-like receptor 4 activation in 2009 (1). Topically, it is also of interest to mention that binding of TNC to HIV has been shown to facilitate viral neutralization, identifying how milk from breast feeding mothers can protect their infants from infection (2). Moreover, TNC was recently found to be elevated in COVID19 patients, specifically those with severe symptoms (3), thus opening opportunities to examine a novel role for TNC in immunity upon viral infection.

We are delighted that in this issue, in 5 short reviews, researchers who were amongst those center stage in the discoveries of the respective tenascin family members set out their views on the early days of tenascin work. These historical perspectives are written by Sakakura and Chiquet (TNC), Rathjen and Hodge (TNR), Miller (TNX) and Degen et al.

This glance into the past, at the very beginning of our examination of the extracellular matrix, reveals the discovery of TNC, the first tenascin family member to emerge, and also the first matrix molecule to be sequenced. TNC was named after its presence in developing (nascent) tissues and at places with physical tension (tendons). Depending on its discovery it received different names such as GMEM (glial mesenchymal extracellular matrix, 1983) by Boudon and collaborators (4), “myotendinous antigen” (1984) by Chiquet and colleagues (5), “hexabrachion” (1984) by Erickson and Ingelsias (6), “Cytotactin” (1985) by Grumet and colleagues (7), before R. Chiquet-Ehrismann coined the term tenascin (1986) (8), that then turned into tenascin-C (1993) (9). The smallest family member, TNR, was first identified in the late 1980s as an axonal associated molecule, and was called restrictin by Rathjen and colleaugues, due to its very specific localization not only within the CNS and spinal cord but also its expression pattern in discrete niches of the neural system (10) or J1-160/180 by Schachner and colleagues (5, 11). The discovery of TNX in the late 1980s occurred entirely by serendipity by investigators hunting for the gene that causes congential adrenal hyperplasia. This was eventually mapped to the *CYP21A2* gene, encoding steroid 21-hydroxylase, but which locus was overlapped by an unknown gene, gene X (12). Upon comparison with existing TNC cDNA sequences, gene X soon became tenascin-X, and its archetypal domain organization and oligomerization established, alongside a key role in dermal collagen organization, and beyond. With unification across these three molecules, and phylogenetic confirmation of tenascin-Y as the chicken homolog of tenascin-X, this triad of the tenascin family was born (13). It was not until 1998, that the baby of the family, TNW, came along. Often cited as the least understood tenascin, TNW was identified and eponymously named by Weber and colleagues using a Zebra fish cDNA library to screen for tenascin related molecules (14), with the mammalian homolog, named TNN, identified in mice by Niehardt et al. in 2003 (15). Confusion, likely caused by different numbers of alternatively spliced domains in TNW and TNN, and lack of consensus around the expression of this molecule made this a difficult birth, and still to date little is known about the biological role of TNW, making it arguably the most intriguing family member.

These perspectives also touch on the generation of the first tenascin knockout animals; key tools in the field, but with an interesting history. For example, the TNC knockout mouse was generated 3 times independently (16–18) as it was hard to reconcile how these animals could be viable and apparently healthy. Although it was clearly shown that tenascins-C, R and W play a role in tissue development and homeostasis (although this is still insufficiently understood) they became more “famous” for the roles they adopted when expressed during disease, rather than in healthy tissue. Knockout mice for each tenascin alone or even in combination (triple knockout for TNC, TNW and TNR (19)) are viable and overtly normal. However closer inspection of both mouse and man revealed that these molecules are not entirely indispensable. For example, deficiency of TNX, and point mutations in TNR and TNC are associated with diseases such as the Ehlers Danlos syndrome (TNX) (20) and neurological aberrations (TNR) (21) and hearing loss and tendinopathy (TNC) (22, 23).

Despite these examples, it is remarkable that the sequence of all tenascins is highly conserved amongst vertebrates suggesting a selection pressure for their maintenance which is not understood at all. In tenascin knockout conditions the different responses to the loss of the respective tenascin protein raises questions about potential compensatory mechanisms. In most knockout mice the respective protein is missing due to deletion of an exon, however most of the remaining mRNA is still expressed. It is intriguing to speculate that the remaining mRNA may have an impact on miR and long non coding (lnc) RNA networks as recently shown for the lncRNA ET20 that plays a role in EMT and was induced by TGFβ. The authors discovered that the ET20 locus is located within the TNC locus in antisense orientation and that ablation of ET20 inhibited TNC expression and TNC-induced EMT (24), altogether opening a new view on how results from knockout mice can be interpreted. It will be important to consider the methodology of tenascin family gene manipulation going forward, and indeed these data may help to interpret other *in vivo* studies.

Here, for the first time, the phenotype of the TNW/TNN knockout mouse is presented. This mouse shows an aberrant tooth (incisor) developmental phenotype amongst other deficits, described by Imhof et al. TNN/TNW deficiency affects periodontal remodeling and increases nerve fiber branching *via* sonic hedghog signaling, presumably increasing pain that led to reduced food intake and lower body weight. In contrast to knockout generation, in this Research Topic, Hendaoui et al. showed over expression of TNW in extrahepatic cancers of the billiary system and propose high expression TNW as potential means to detect this type of cancer earlier that stays too long asymptomatic showing already metastasis at the time of diagnosis. Moreover, in this Research Topic, new insights into the role of TNC are reviewed, including in joint disease (Hasegawa et al.), myocarditis/cardiomyopathy (Tajiri et al.), glaucoma (Wiemann et al.), sepsis (Meijer et al.), stroke (Okada and Suzuki), glial scar formation (Bijelić et al.) and pancreatic cancer (Liot et al.).

All tenascins have context dependent functions. This has clearly been shown for TNC in joint disease. Of note in this Research Topic the role of TNC as DAMP in rheumatoid arthritis (RA) and as a regulator of tissue repair in osteoarthritis (OA) is discussed by Hasegawa et al., who emphasize the importance of considering the context specific action of this matrix molecule in different disease settings even within the same tissue. More recently, pro- and anti-inflammatory roles for TNC have been reported in cancer (25–29) and it is clear that we need good *in vivo* models to better understand the functions of tenascins in space and time, and in a cell type specific manner. Four novel murine models have been described here; a sepsis model using Klebsiella in wildtype and TNC knockout animals, revealing a moderately lower bacterial load in lungs and blood, that does not translate to impact on severity of the disease symptoms (Meijer et al.), the first inducible TNC over expression mouse ectopically expressing TNC in heart, increasing expression of inflammatory cytokines and increasing mortality during the acute stage after myocardial infarction (Yonebayashi et al.), a model of autoimmune glaucoma (Wiemann et al.) and a novel orthotopic tongue squamous cell carcinoma cell grafting model that is sensitive to radiotherapy (Spenlé et al.).

In search of the underlying mechanisms of tenascin action, attention has focused on the different proteoforms of these extensively alternatively spliced genes. Iyoda et al. review results on the generation and actions of the alternatively spliced, and cryptic, TNIIIA2 domain in the malignancy of glioblastoma (GBM). This involves MMP9 secretion by TNC-activated macrophages generating TNIIIA2 from intact TNC, activating β1 integrin signaling in conjunction with syndecan-4 enhancing cell adhesion and malignancy of GBM cells. In this Research Topic, Bijelić et al. used recombinantly expressed proteins representing different domains of TNC to interrogate regulation of astrocyte behaviour *in vitro* and *in vivo*. *In vitro*, TNC fragments induced pro-inflammatory cytokine production. Alternatively spliced TNIIID, TNIIIA and their combination strongly decreased proliferation and delayed gap closure of scratch wound assay of cultured astrocytes. *In vivo*, TNIIID or TNIII(D+A) led to higher expression of GFAP in the wild type mice than TNC knockout mice. Addition of TNIIID to TNC knockout mice increased the activated microglia although overall cell proliferation in injured sites were not affected. Aubert et al. focus on another domain, conserved in all family members and revealed a TGFβ pathway-promoting function of the FBG domain of all four tenascins, where binding of the latent form of TGFβ and its activation are addressed. These results are intriguing and reveal that in addition to thrombospondin 1, connective tissue factor (CTGF, CCN2) and previously shown TNX also the FBG of TNC, TNW and TNR can bind TGFβ and activate TGFβ receptor signaling. Finally, Albacete-Albacete et al. review the transportation of ECM components by extracellular vesicles. Exosomal secretion is particularly critical for extracellular release and deposition of TNC (30) and has been shown to follow rhinovirus infection of epithelial airways (31). Circulating exosomes from cancer patients frequently carry TNC and exosomal TNC is fully functional, which may induce a proinflammatory state and contribute to premetastatic niche formation.

While understanding the roles of a particular tenascin requires models that enable correlation between phenotype to high and no expression of the respective tenascin, it is obvious that tenascins form networks with other molecules which may define their highly context dependent actions. In this Research Topic, Liot et al. summarize the roles of TNC in context of other known matrisomal molecules impacting disease severity of pancreatic ductal adenocarcinoma. Okada and Suzuki review literature on TNC-induced roles on inflammation in context of other matrix molecules on stroke-related pathologies while Matsumoto and Aoki review the roles of TNC in conjunction with TNX in the cardiovascular system. TNX and TNC have distinct roles in physiological and pathological conditions. TNX is involved in the structural integrity of collagen fibrils, activation and its absence causes classical-like Ehlers-Danlos syndrome. In contrast, the role of TNC can be detrimental or beneficial also in a context-dependent manner. TNC may prevent aortic dissection and rupture of cerebral aneurysm, while it may exacerbate acute vasospastic response and cerebral injury.

Finally, it has been evident for some time now that tenascins may be reliable biomarkers for disease diagnosis and targets for new therapies. Dhaouadi et al. developed nanobodies against TNC and showed binding in the central constant TNIII domains of the molecule. These nanobodies recognized human and murine TNC in paraffin embedded tumor tissue (useful for the clinical practice) and blocked cell rounding and chemoretention of dendritic cells by TNC potentially thus being valuable for therapy. Other nanobodies specific for TNC have recently been proposed to be also valuables tools in particular for imaging tumors (US 2019/0225693 A1 patent by RO Hynes).

Altogether despite the discovery of TNC more than 4 decades ago, followed by the identification and characterization of the other 3 family members, it is clear that we still are at the beginning of our understanding of the molecular and structural networks in which the respective tenascin molecule is expressed and what each member is doing there. Moreover, as all members can be regulated by splicing and modified by glycosylation more research has to be done to identify which proteoform is expressed when and where and what roles these different molecules have. Novel tools and a broader understanding of tenascin expression and functions as described in this Research Topic are valuable and may help to prepare a future application of this knowledge in diagnosis and therapy.

## Author Contributions

All authors listed have made a substantial, direct, and intellectual contribution to the work and approved it for publication.

## Funding

GO was supported by ANR-MatrixNash, INCa-PLBIO TENMAX, Aviesan Radio 3R, Ligue contre le Cancer CCIRGE.

## Conflict of Interest

The authors declare that the research was conducted in the absence of any commercial or financial relationships that could be construed as a potential conflict of interest.

## Publisher’s Note

All claims expressed in this article are solely those of the authors and do not necessarily represent those of their affiliated organizations, or those of the publisher, the editors and the reviewers. Any product that may be evaluated in this article, or claim that may be made by its manufacturer, is not guaranteed or endorsed by the publisher.
